# Ability of SLE-DAS and SLEDAI-2K to predict damage accrual in SLE: a real-world 7-year cohort study from Sweden

**DOI:** 10.1093/rheumatology/keag545

**Published:** 2026-09-29

**Authors:** Rama Andraos, Alexander Tsoi, Alf Kastbom, Martina Frodlund, Diogo Jesus, Luís Sousa Inês, Ioannis Parodis, Christopher Sjöwall

**Affiliations:** Division of Inflammation and Infection/Rheumatology, Department of Biomedical and Clinical Sciences, Linköping University, Linköping, Sweden; Division of Rheumatology, Department of Medicine Solna, Karolinska Institutet, Karolinska University Hospital, and Center for Molecular Medicine (CMM), Stockholm, Sweden; Division of Inflammation and Infection/Rheumatology, Department of Biomedical and Clinical Sciences, Linköping University, Linköping, Sweden; Division of Inflammation and Infection/Rheumatology, Department of Biomedical and Clinical Sciences, Linköping University, Linköping, Sweden; Rheumatology Department, ULS Região de Leiria, Leiria, Portugal; Faculty of Health Sciences, University of Beira Interior, Covilhã, Portugal; Faculty of Health Sciences, University of Beira Interior, Covilhã, Portugal; Rheumatology Department, Hospital da Universidade de Coimbra—Unidade Local de Saúde de Coimbra, Coimbra, Portugal; Faculty of Medicine, University of Coimbra, Coimbra, Portugal; Division of Rheumatology, Department of Medicine Solna, Karolinska Institutet, Karolinska University Hospital, and Center for Molecular Medicine (CMM), Stockholm, Sweden; Department of Rheumatology, Faculty of Medicine and Health, Örebro University, Örebro, Sweden; Division of Inflammation and Infection/Rheumatology, Department of Biomedical and Clinical Sciences, Linköping University, Linköping, Sweden

**Keywords:** systemic lupus erythematosus, disease activity, organ damage, SLE-DAS, SLEDAI-2K

## Abstract

**Objectives:**

SLE remains a heterogeneous autoimmune disease that can lead to irreversible organ damage. SLE Disease Activity Score (SLE-DAS) is a recently developed tool with higher sensitivity to change than SLE Disease Activity Index-2000 (SLEDAI-2K). We compared SLE-DAS with other activity measures in terms of ability to predict organ damage.

**Methods:**

Patients from Linköping University Hospital with ≥2 visits between 2008 and 2025 were included. SLEDAI-2K and Physician’s Global Assessment (PhGA) were assessed prospectively, and SLE-DAS retrospectively. Organ damage was estimated annually using the SLICC/ACR Damage Index (SDI). Adjusted mean scores of SLEDAI-2K (amSLEDAI-2K), PhGA (amPhGA) and SLE-DAS (amSLE-DAS) were determined for each time interval between consecutive visits, and their ability to predict damage accrual was evaluated using time-dependent multivariable Cox regression models. Predictive ability was compared across measures using Akaike Information Criterion (AIC) and Bayesian Information Criterion (BIC).

**Results:**

In total, 335 patients with 3582 outpatient visits contributed data. amSLE-DAS was an independent predictor of SDI increase at the 3- (HR; 95% CI: 1.04; 1.00–1.08), 5- (1.05; 1.01–1.09) and 7-year follow-up (1.04; 1.00–1.08). amSLEDAI-2K was predictive at the 5-year follow-up only (1.12; 1.02–1.22), and amPhGA at 5 (1.99; 1.19–3.36) and 7 (1.84; 1.02–3.30) years. amSLE-DAS showed a higher predictive ability for damage accrual than amSLEDAI-2K at the 3-, 5- and 7-year follow-up, as indicated by lower AIC and BIC.

**Conclusion:**

In this real-world Swedish setting, we demonstrate that SLE-DAS outperforms SLEDAI-2K in predicting organ damage accrual in SLE, substantiating its clinical relevance.

*Rheumatology* key messages

- Longitudinal SLE-DAS assessment effectively predicted organ damage accrual in a real-world SLE setting.
- SLE-DAS outperformed SLEDAI-2K in predicting organ damage progression, especially early during the SLE course.
- In line with prior validation studies, SLE-DAS demonstrated ability to capture clinically meaningful information.

## Introduction

The clinical manifestations of systemic lupus erythematosus (SLE) exhibit marked heterogeneity, from mild disease limited to cutaneous and/or musculoskeletal involvement to severe, life-threatening presentations characterized by kidney affliction, profound autoimmune cytopaenias, central nervous system complications and thromboembolic events [1, 2]. The management of patients with SLE should prioritize attainment of remission and prevention of flares and accrual of irreversible organ damage [3]. Disease activity, assessed by validated instruments, is prognostic for organ damage accrual, which in turn is a major determinant of long-term patient prognosis and survival [3–5]. Accurate evaluation of SLE disease activity using validated instruments is recommended to monitor patient status, determine prognosis and guide therapeutic decision-making [6]. Several instruments have been developed for this purpose, including the SLE Disease Activity Index-2000 (SLEDAI-2K), Systemic Lupus Activity Measure (SLAM) and British Isles Lupus Assessment Group (BILAG) index [7, 8].

Each of these legacy assessment tools exhibits intrinsic limitations for their usefulness in the context of daily clinical practice. The SLEDAI-2K offers ease of use and practicality; however, its reliance on strictly dichotomous scoring restricts its ability to reflect gradations in disease activity within individual organ manifestations [9]. In addition, SLEDAI-2K does not include important features such as haemolytic anaemia or cardiopulmonary involvement, thereby diminishing its sensitivity to change [10, 11]. These information gaps and imbalances in weighting of manifestations in SLEDAI-2K, such as attributing the same score to either systemic or cutaneous vasculitis, limit its accuracy for measuring global disease activity. Conversely, the BILAG index affords a more nuanced and comprehensive evaluation of organ-based disease activity, yet its complexity and high administrative burden constrain its feasibility in routine clinical practice [8]. Furthermore, the original BILAG index was never designed to measure global disease activity [9].

To overcome these limitations, the SLE Disease Activity Score (SLE-DAS) is a recently developed instrument for the assessment of disease activity in SLE, which has demonstrated robust validity, reliability, accuracy in measuring global disease activity and sensitivity to change over time across diverse clinical settings [12, 13]. SLE-DAS is derived from a weighted algorithm encompassing 17 parameters, comprising both dichotomous variables, e.g. hemolytic anemia, cardiac involvement, and cutaneous manifestations, and continuous variables, including arthritis, proteinuria, leukopenia and thrombocytopaenia [14]. To facilitate its clinical application, a web-based calculator (http://sle-das.eu/) enables scoring and interpreting the SLE-DAS in about 1–2 min. To date, SLE-DAS has been validated in cohorts from Southern Europe, and data from Asia and Latin America are emerging, but no validation has been conducted in Northern European populations [15–19]. Implementation of SLE-DAS in routine clinical practice and as an outcome measure in clinical trials requires its broad validation as an instrument that provides greater discriminatory capacity to detect clinically meaningful changes in disease activity and predict organ damage than alternative instruments [12].

In this study, we aimed to assess the ability of SLE-DAS to predict organ damage accrual and compare it with other SLE disease activity instruments in a Swedish longitudinal real-world cohort. Furthermore, subgroup analyses were performed according to relevant clinical characteristics, i.e. history of or current renal, hematological and articular involvement.

## Methods

### Study population

The *Clinical Lupus Register in the North-eastern Gothia* (Swedish acronym: KLURING) cohort was established in early 2008 and has enrolled, and longitudinally followed, all adult patients with SLE in Region Östergötland, Sweden (*N *= 358 at the time of data extraction) [20]. All participants had a clinical diagnosis of SLE and met the 1982 American College of Rheumatology (ACR) and/or the 2012 Systemic Lupus International Collaborating Clinics (SLICC) classification criteria [21]. For the current study, we selected all patients from the KLURING cohort who had contributed with data from at least two visits. This resulted in a dataset comprising 335 of 358 available subjects (94%) and 3582 outpatient visits.

### Assessments of disease activity, remission and organ damage

Disease activity was assessed at each visit using SLEDAI-2K, clinical (c)SLEDAI-2K (items for dsDNA binding and complement consumption being excluded) and the Physician’s Global Assessment (PhGA; scale: 0–3) [7]. Remission was recorded according to *Definition Of Remission In SLE* (DORIS) [3]. Organ damage was quantified annually since the launch of KLURING using the SLICC/ACR damage index (SDI) [22]. SLE-DAS was retrospectively calculated in 2025, using prospectively collected clinical and laboratory data from each visit. All calculations were performed by the same rheumatologist (R.A.).

The time-adjusted mean score of SLEDAI-2K (amSLEDAI-2K), cSLEDAI-2K (amcSLEDAI-2K), PhGA (amPhGA) and SLE-DAS (amSLE-DAS) were determined for each patient and each time interval between consecutive visits [12, 23]. The ability of each instrument to predict organ damage accrual (defined as any SDI increase) was evaluated in a time-dependent manner.

Laboratory monitoring performed at the Linköping University Hospital included assessment of complete blood counts, erythrocyte sedimentation rate (ESR) and plasma levels of C-reactive protein (CRP), creatine kinase (CK) and creatinine for calculation of the estimated glomerular filtration rate (eGFR), as well as assessment of complement protein C3 and C4 levels, anti-dsDNA antibodies and urinalysis, including urinary sediment and quantification of proteinuria.

### Statistics

The ability of amSLEDAI-2K, amcSLEDAI-2K, amPhGA and amSLE-DAS to predict organ damage accrual (increase in SDI ≥1) was evaluated using multivariable Cox regression models. Models were adjusted for age, sex and baseline SDI score (fixed covariates), and glucocorticoid dose (estimated as mg/day of prednisone equivalents) as a time-dependent variable, following the approach used in the SLE-DAS derivation and validation study [12]. Models were applied for 3, 5 and 7 years of follow-up. The predictive performance of amSLEDAI-2K, amcSLEDAI-2K and amSLE-DAS were compared using Akaike Information Criterion (AIC) and Bayesian Information Criterion (BIC).

AIC and BIC are widely used measures for comparing and selecting statistical models. Both assess model fit while penalizing model complexity to reduce the risk of overfitting. In regression analyses, they are particularly useful for comparing models with different combinations of predictors or confounders. Lower AIC and BIC values indicate a better balance between goodness of fit and model complexity and are therefore generally preferred. Although both criteria serve a similar purpose, BIC imposes a stronger penalty for additional model parameters, particularly in larger datasets, whereas AIC applies a constant penalty and is generally more focused on predictive performance. Since SLEDAI-2K and SLE-DAS differ in how some items are scored (dichotomous *vs* continuous), we conducted subgroup analyses for subjects meeting criteria for arthritis, renal and haematological disorders (according to the 1982 ACR definitions) [24]. The Spearman’s correlation coefficient was employed to examine relationships between the various SLE disease activity instruments.

Differences yielding *P*-values <0.05 were considered statistically significant. SPSS Statistics V.31.0.1.0 (IBM Corp., Armonk, NY, USA) and the R-4.5.2 software (R Foundation for Statistical Computing, Vienna, Austria) were used for statistical analyses and generation of forest plots, and the GraphPad Prism Version 10.6 (GraphPad Software, Boston, MA, USA) was used for the generation of other figures.

### Ethics approval

The study was conducted according to the Declaration of Helsinki, and the study protocol was approved by the Regional Ethics Board in Linköping (M75-08/2008).

## Results

The study cohort included 335 patients, of whom 85 (25.4%) had current or prior lupus nephritis (LN). Of the 335 patients in total, 289 (86.3%) were women, with main study population characteristics detailed in Table 1. The proportions of patients attaining DORIS remission over time are demonstrated in Supplementary Table S1.

**Table 1:** Detailed characteristics of patients included in the study.

	All	%	Women	%	Men	%
Patients, *N*	335	100	289	86.3	46	13.7
Caucasian ethnicity, *N*	292	87.2	248	85.8	44	95.6
Included in cohort as incident SLE, *N*	130	38.8	104	36.0	26	56.5
Outpatient visits, *N*	3582	100	3164	88.3	418	11.7
Age at SLE diagnosis, *years* (mean ± SD)	40 (17.6)	–	39 (16.4)	–	49 (22.1)	–
SLE duration, *years* (median ± IQR)	4.1 (12.9)	–	5.1 (13.0)	–	1.1 (8.4)	–
SLE-DAS, *score* (median ± IQR)	1.3 (6.8)	–	1.3 (5.8)	–	1.3 (14.1)	–
SLEDAI-2K, *score* (median ± IQR)	2 (5)	–	2 (4)	–	2 (6)	–
PhGA (median ± IQR)	0 (1)	–	0 (1)	–	0 (1)	–
SDI at baseline, *score* (median ± IQR)	0 (1)	–	0 (1)	–	0 (1)	–
Baseline prednisone-equivalent dose, *mg/day* (median ± IQR)	5.0 (7.5)	–	5.0 (7.5)	–	5.0 (6.6)	–
Antimalarial therapy, *N*	234	69.9	206	71.3	28	60.9
Conventional immunosuppressants, *N*	108	32.2	88	30.4	20	43.5
Biological therapy, *N*	23	6.9	20	6.9	3	6.5
Tobacco smoking at baseline (ever/ongoing), *N*	147	43.9	124	42.9	23	50.0
**Lab at baseline**						
ESR, *mm/h* (median ± IQR)	14.0 (20.8)	–	14.0 (18.3)	–	13.5 (27.0)	–
CRP, *mg/L* (median ± IQR)	3.2 (5.6)	–	2.7 (5.9)	–	5.0 (12.7)	–
CK, *µkatal/L* (median ± IQR)	1.3 (1.0)	–	1.2 (1.1)	–	1.4 (0.8)	–
Creatinine, *µmol/L* (mean ± SD)	74.5 (29.6)	–	70.0 (19.8)	–	102.8 (55.0)	–
eGFR^a^, *mL/min/1.73m²* (mean ± SD)	86.6 (30.5)	–	87.8 (30.6)	–	79.0 (29.1)	–
Complement protein 3, *g/L* (mean ± SD)	0.97 (0.27)	–	0.96 (0.27)	–	1.03 (0.29)	–
Complement protein 4, *g/L* (mean ± SD)	0.18 (0.10)	–	0.17 (0.10)	–	0.20 (0.11)	–
Positive anti-dsDNA antibody, *N*	161	48.1	140	48.4	21	45.6
**Fulfilled 1982 ACR criteria**						
ACR-1: malar rash	122	36.4	115	39.8	7	15.2
ACR-2: discoid lupus	49	14.6	43	14.9	6	13.0
ACR-3: photosensitivity	171	51.0	157	54.3	14	30.4
ACR-4: oral ulcers	47	14.0	42	14.5	5	10.9
ACR-5: arthritis	260	77.6	224	77.5	36	78.3
ACR-6: serositis	119	35.5	97	33.6	22	47.8
ACR-7: renal disorder	85	25.4	65	22.5	20	43.5
ACR-8: neurologic disorder	21	6.3	18	6.2	3	6.5
ACR-9: hematologic disorder	210	62.7	177	61.2	33	71.7
ACR-10: immunologic disorder	189	56.4	160	55.4	29	63.0
ACR-11: antinuclear antibody	331	98.9	285	98.6	46	100

a Modification of diet in renal disease was used as formula for estimating glomerular filtration rate.

Abbreviations: ACR, American College of Rheumatology; IQR, interquartile range; PhGA, Physician’s Global Assessment; SD, standard deviation; SDI, SLICC/ACR damage index; SLE, systemic lupus erythematosus; SLEDAI-2K, SLE disease activity index-2000; SLE-DAS, SLE disease activity score.

Using data from all 3582 outpatient visits, SLE-DAS scores were strongly correlated with SLEDAI-2K (rho = 0.73, *P* < 0.001) and moderately with cSLEDAI-2K (rho = 0.54, *P* < 0.001) and PhGA (rho = 0.45, *P* < 0.001). These correlations remained statistically significant across all subgroups of interest, i.e. men and women assessed separately, patients with and without a history of LN and patients enrolled in KLURING at the timepoint of SLE diagnosis (inception cohort). Slightly weaker correlations were found among men, especially those without a history of LN (Fig. 1).

**Figure 1:**
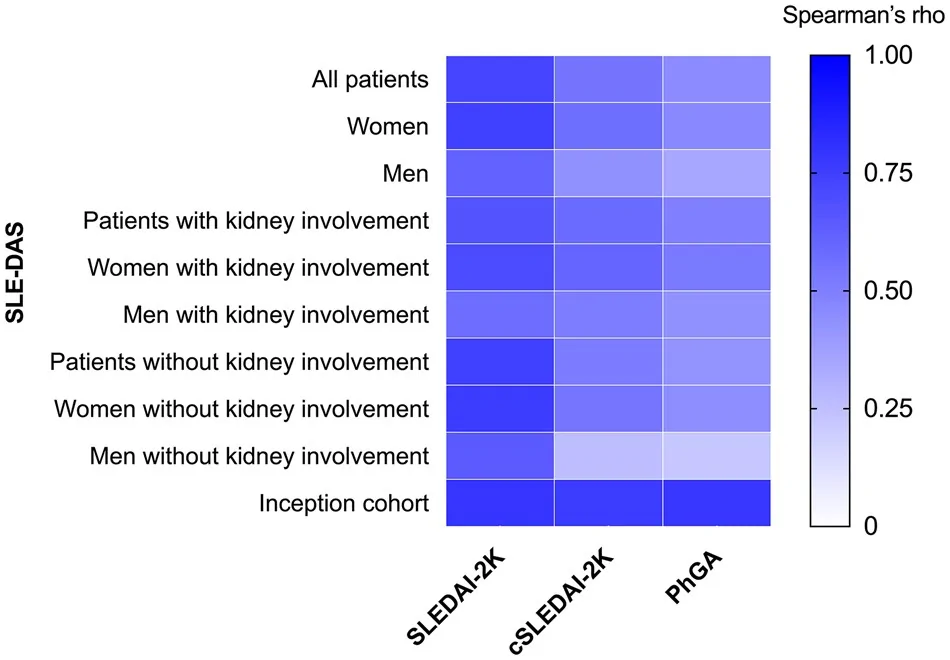
Associations between SLE-DAS and other disease activity assessments. Correlations are shown by heat-mapping Spearman’s correlation coefficients. All correlations are statistically significant at the level of *P* < 0.001. Abbreviations: SLE-DAS, SLE Disease Activity Score; cSLEDAI-2K, clinical SLE Disease Activity Index-2000; SLEDAI-2K, SLE Disease Activity Index-2000; PhGA, Physician’s Global Assessment

For the analysis of prediction of organ damage accrual with adjusted mean scores, data were evaluated for the full cohort (*N *= 335) and for subgroups of interest defined by the 1982 ACR criteria: history of LN (*N *= 85), arthritis (*N *= 260) and haematological disorder (*N *= 210). Results are presented for all available visits as well as for the 3-, 5- and 7-year follow-up. All models were adjusted for baseline SDI score, age, sex and daily prednisone equivalent dose.

In the full cohort, amSLE-DAS was a statistically significant predictor of damage progression at the 3-, 5- and 7-year follow-up (*P* = 0.030, *P* = 0.013 and *P* = 0.048, respectively). amPhGA predicted damage at year 5 (*P* = 0.009) and year 7 (*P* = 0.042). amSLEDAI-2K was a statistically significant predictor of organ damage progression only at the 5-year follow-up (*P* = 0.019) (Fig. 2). None of the indices predicted organ damage accrual when all available visits (including visits beyond 7 years) were considered in the models. amSLE‑DAS demonstrated greater predictive value for organ damage accrual than amSLEDAI-2K at the 3‑, 5‑ and 7‑year follow‑up, as reflected by lower AIC and BIC values (Supplementary Table S2).

**Figure 2:**
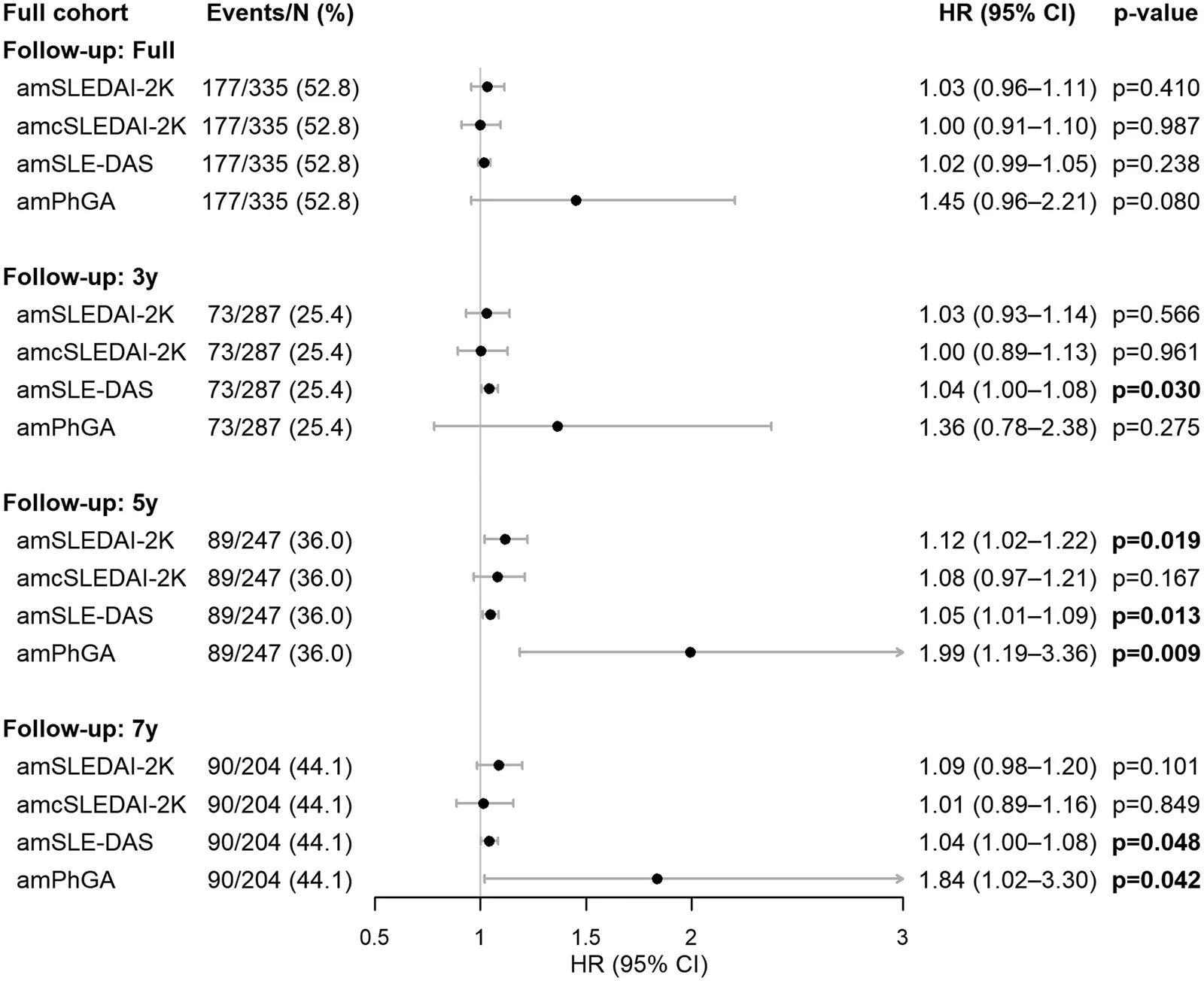
Multivariable Cox regression analysis for damage accrual across all available visits. Forest plot showing HR with 95% CI for four time-varying disease activity measures stratified by follow-up duration. Models are adjusted for baseline SDI, age, sex and mean glucocorticoid dose. Abbreviations: amSLEDAI-2K, adjusted mean SLE Disease Activity Index-2000; amcSLEDAI-2K, adjusted mean clinical SLEDAI-2K; amSLE-DAS, adjusted mean SLE-DAS; amPhGA, adjusted mean Physician’s Global Assessment; CI, confidence interval; HR, hazard ratio; SDI, SLICC/ACR Damage Index

In the LN subgroup, both amSLE‑DAS (*P* = 0.032) and amPhGA (*P* = 0.019) were significant predictors of organ damage accrual at 3 years, and amPhGA remained predictive also at the 5‑year follow‑up (*P* = 0.014; Fig. 3). In participants meeting the arthritis ACR criterion, amPhGA was a significant predictor of organ damage accrual at the 5- and the 7-year follow‑up as well as in the analysis of all available visits (*P* = 0.031, *P* = 0.002 and *P* = 0.013, respectively), whereas amSLEDAI-2K yielded statistical significance at the 5-year follow-up (*P* = 0.013) only (Fig. 4).

**Figure 3:**
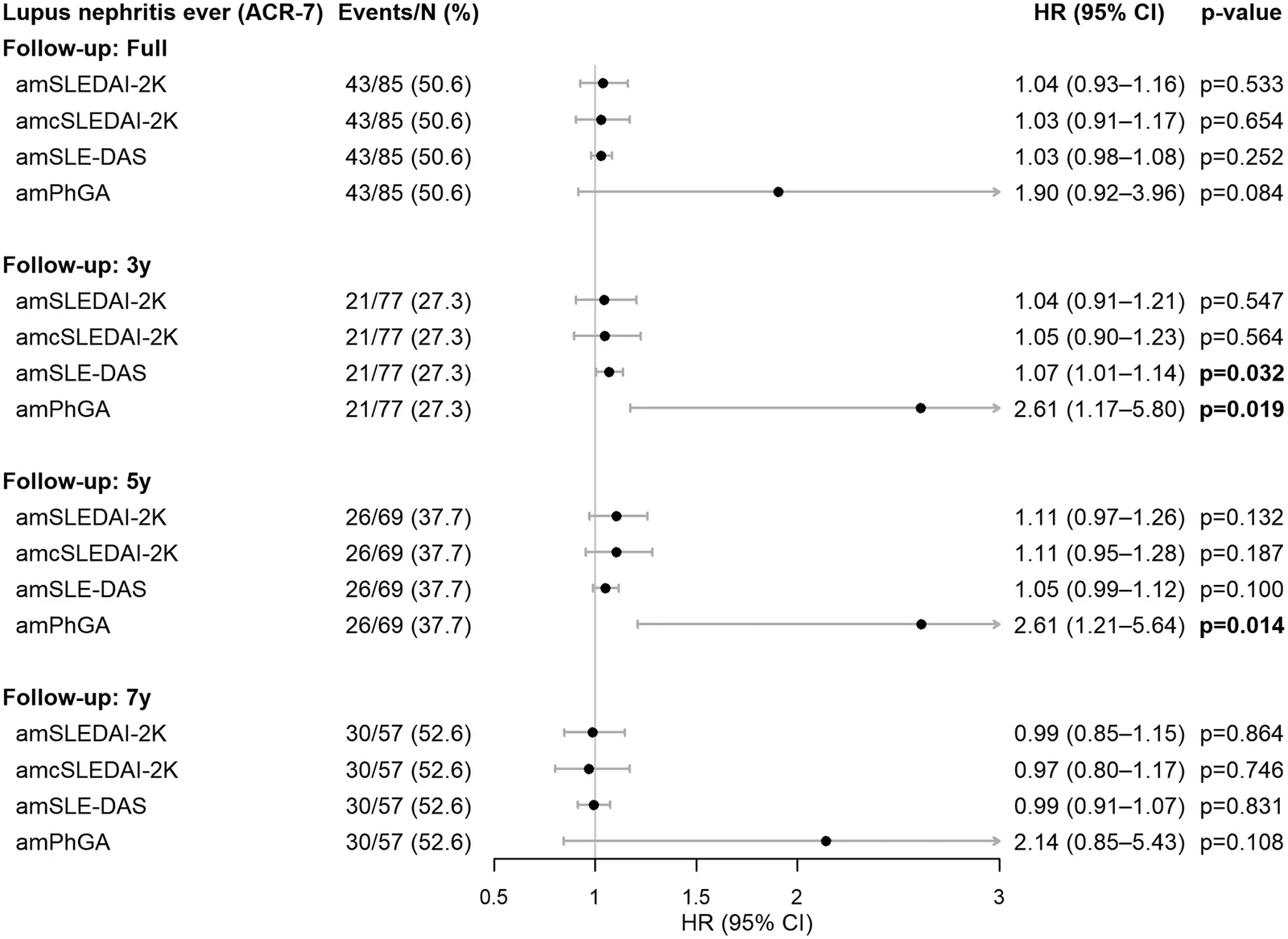
Multivariable Cox regression analysis for damage accrual in patients with a history of lupus nephritis (ACR criterion 7). Forest plot showing HRs with 95% CIs for four time-varying disease activity measures stratified by follow-up duration. Models are adjusted for baseline SDI, age, sex and mean glucocorticoid dose. Abbreviations: ACR, American College of Rheumatology; amSLEDAI-2K, adjusted mean SLE Disease Activity Index-2000; amcSLEDAI-2K, adjusted mean clinical SLEDAI-2K; amSLE-DAS, adjusted mean SLE-DAS; amPhGA, adjusted mean Physician’s Global Assessment; CI, confidence interval; HR, hazard ratio; SDI, SLICC/ACR Damage Index

**Figure 4:**
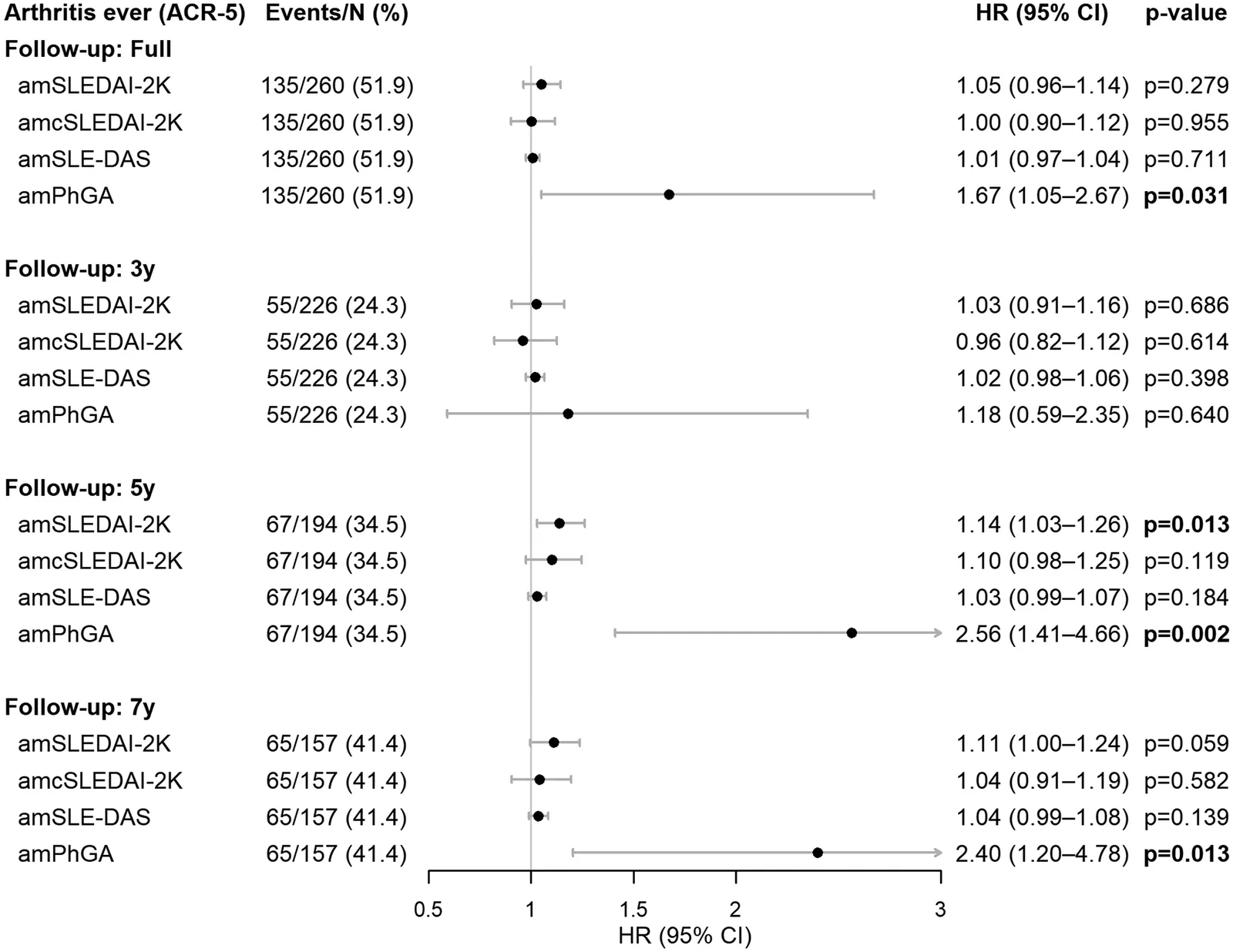
Multivariable Cox regression analysis for damage accrual in patients with a history of arthritis (ACR criterion 5). Forest plot showing HRs with 95% CIs for four time-varying disease activity measures stratified by follow-up duration. Models are adjusted for baseline SDI, age, sex and mean glucocorticoid dose. Abbreviations: ACR, American College of Rheumatology; amSLEDAI-2K, adjusted mean SLE Disease Activity Index-2000; amcSLEDAI-2K, adjusted mean clinical SLEDAI-2K; amSLE-DAS, adjusted mean SLE-DAS; amPhGA, adjusted mean Physician’s Global Assessment; CI, confidence interval; HR, hazard ratio; SDI, SLICC/ACR Damage Index

For subjects with haematological disorder, amSLE-DAS was a statistically significant predictor of organ damage accrual at the 3-, 5- and 7-year follow-up (*P* = 0.029, *P* = 0.010 and *P* = 0.011, respectively), whereas amSLEDAI-2K and amPhGA predicted SDI increase at the 5-year (*P* = 0.004 and *P* = 0.008, respectively), and the 7-year (*P* = 0.025 and *P* = 0.014, respectively) follow-up (Fig. 5).

**Figure 5:**
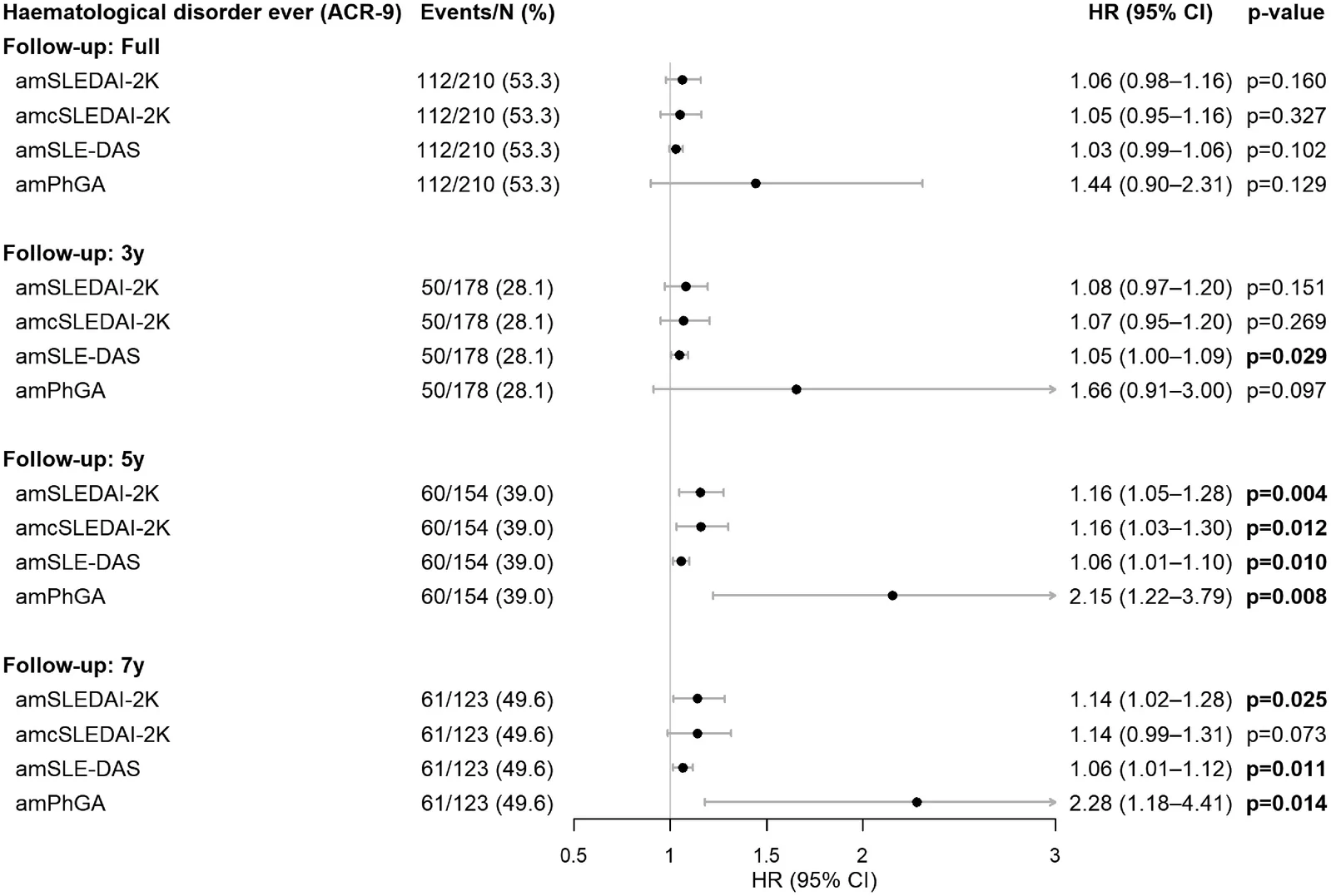
Multivariable Cox regression analysis for damage accrual in patients with a history of haematologic disorder (ACR criterion 9). Forest plot showing HRs with 95% CIs for four time-varying disease activity measures stratified by follow-up duration. Models are adjusted for baseline SDI, age, sex and mean glucocorticoid dose. Abbreviations: ACR, American College of Rheumatology; amSLEDAI-2K, adjusted mean SLE Disease Activity Index-2000; amcSLEDAI-2K, adjusted mean clinical SLEDAI-2K; amSLE-DAS, adjusted mean SLE-DAS; amPhGA, adjusted mean Physician’s Global Assessment; CI, confidence interval; HR, hazard ratio; SDI, SLICC/ACR Damage Index

## Discussion

The main finding of the current study is that SLE-DAS conveys clinically meaningful information and provides superior performance compared with SLEDAI-2K in predicting damage accrual in real-world settings, particularly among patients with a SLE phenotype including haematological manifestations.

Preventing organ damage has become a central goal of contemporary SLE management [6, 25, 26]. This emphasis is reflected in the inclusion of the SDI as an endpoint in recent randomized controlled trials [27–29]. This study was conducted to evaluate the capacity of selected SLE disease activity assessment instruments to predict organ damage accrual. The evaluated disease activity assessment instruments demonstrated a high degree of correlation, from moderate to strong, with each other. In multivariable Cox regression analyses, amSLE-DAS emerged as an independent predictor of organ damage accrual at the 3-, 5- and 7-year follow-up assessments. In contrast, amSLEDAI-2K demonstrated predictive value at the 5-year follow-up only. These observations are in line with earlier validatory work for SLE-DAS and illustrate that scoring SLE-DAS appears to be clinically meaningful and superior or at least comparable to SLEDAI-2K in predicting organ damage accrual [12, 18].

Given that SLEDAI-2K and SLE-DAS apply dichotomous *vs* continuous scoring for certain items, subgroup analyses were undertaken among subjects fulfilling the arthritis, renal and haematological disorder criteria of the 1982 ACR classification [24]. Intuitively, amSLE-DAS showed particularly strong predictive performance among patients with a history of haematological involvement. Across all evaluated time points (3, 5 and 7 years of follow-up), amSLE-DAS consistently exhibited superior predictive ability compared with amSLEDAI-2K, as reflected by lower AIC and BIC values.

Importantly, SLEDAI-2K and PhGA were assessed contemporaneously at clinical visits, potentially influencing subsequent therapeutic decisions, whereas SLE-DAS was calculated retrospectively for the purpose of the present study. Thus, the physicians at each visit were blinded for the score of SLE-DAS, but not for SLEDAI-2K, cSLEDAI-2K or PhGA. However, the extent to which differences in the timing of data collection have affected the prognostic value of longitudinal changes in SDI remains unclear.

Over the years, numerous instruments have been developed to assess disease activity in SLE, reflecting both ongoing efforts to improve measurement accuracy and the recognition that no single tool is entirely comprehensive or sufficiently user-friendly [7, 9]. At the same time, this proliferation of assessment tools highlights the inherent complexity of SLE, a multisystem disease in which evaluating disease activity is often complicated by overlapping clinical manifestations, and challenges in attributing findings specifically to SLE rather than to comorbidities or treatment-related side-effects [8].

Ethnic origin and race are among well-recognized determinants of the heterogeneous nature and course of SLE, frequently contributing to divergent clinical findings and outcomes across studies [30]. Previous investigations employing the SLE-DAS have primarily included populations from Southern Europe, as well as cohorts comprising patients of Asian and Hispanic ancestry [12, 15, 17, 18]. To our knowledge, the present study represents the first evaluation of SLE-DAS in a Northern European cohort, in which ∼90% of participants were of Caucasian origin. One study conducted in a Latin American cohort reported that SLE-DAS is a useful instrument for measuring disease activity in SLE [15]. However, the authors found no additional advantage over SLEDAI-2K, and the performance of SLE-DAS appeared limited in a subgroup of 60 patients with high disease activity. In contrast, a recent study from Brazil concluded that SLE-DAS is valid and capable of adequately measuring SLE disease activity [31]. Similarly, a single-centre retrospective study from China suggested that SLE-DAS may offer a more precise assessment of disease activity, particularly in patients with severe thrombocytopenia and cardiopulmonary involvement [18].

Overall, SLE-DAS provided the most precise prediction of subsequent organ damage accrual in our study. While the estimated hazard ratios (HR) were numerically greater for the PhGA, differences in the underlying measurement scales preclude direct comparison of the corresponding effect size. It is also plausible that PhGA rating is more closely linked to treatment decisions, which may partially explain its association with organ damage accrual. While the implementation of PhGA for evaluation of SLE disease activity has been debated in literature, it is a widely used tool in clinical practice to express the perspective of the physician [9]. However, despite otherwise stated in recommendations [32], PhGA in routine clinical practice may be completed in an unstandardized manner and be subjectively influenced by factors like pain, fatigue and laboratory test values [33]. Still, PhGA remains an important component of the definitions of both *Lupus Low Disease Activity State* and DORIS remission [3, 34]. Attempts have recently been made to standardize how PhGA is scored [32]. Nevertheless, it is acknowledged that PhGA is inevitably characterized by considerable inter- and intra-observer variability. Despite these limitations, PhGA may capture clinical manifestations not fully represented in other disease activity indices and is strongly influencing the therapeutic decisions made by the treating rheumatologist [35]. Multiple factors may impact PhGA scoring, including laboratory parameters and the results of validated disease activity measures [36]. In the present investigation, it is plausible that PhGA assessments were influenced by SLEDAI-2K scoring at the same visit.

This study has several limitations. First, the time-adjusted mean disease activity measures account for unequal intervals between visits, patients with highly active disease may undergo more frequent assessments and the potential influence of informative follow-up cannot be entirely excluded. Second, although SLEDAI-2K is widely used, numerous other disease activity assessment instruments are employed globally and in randomized controlled trials. In particular, data on BILAG and SLAM were not available in this cohort, precluding direct comparisons of SLE-DAS with these instruments. Hence, implementation of SLE-DAS in routine clinical practice may require additional assessments and comparisons with other instruments. Furthermore, it remains unclear how effectively SLE-DAS or SLEDAI-2K may predict the development of cumulative organ damage using the forthcoming revised SDI [37]. Finally, the findings were derived from a Scandinavian SLE cohort predominantly comprising patients of Caucasian origin and may therefore not be readily generalizable to SLE populations of other ethnic backgrounds. Conversely, a major strength of the study is the well-characterized cohort, which includes comprehensive data and long-term follow-up of nearly all patients with SLE in the geographical area of Region Östergötland, thereby enhancing the robustness and clinical relevance of the findings, especially in Northern European settings of SLE [20]. An additional strength was the use of AIC and BIC for comparisons across disease activity measures, which mitigates the bias otherwise inferred by the differential scales of the indices.

To conclude, in this real-world Swedish cohort, we demonstrate that SLE-DAS outperforms SLEDAI-2K, cSLEDAI-2K and PhGA in predicting organ damage accrual in SLE, further substantiating its clinical relevance. These findings confirm previous validation studies. Nevertheless, additional and desirably larger investigations are needed to further establish the utility of SLE-DAS as a disease activity measure and corroborate its validity across SLE populations worldwide.

## Supplementary Material

keag545_Supplementary_Data

## Data Availability

The datasets used and analyzed during the current study are available from the corresponding author upon reasonable request.

## Notes

*Disclosure statement*: I.P. has received research funding and/or honoraria from Amgen, AstraZeneca, Amgen, Aurinia, Biogen, Bristol-Myers Squibb (BMS), Elli Lilly, Gilead, GlaxoSmithKline (GSK), Janssen, Novartis, Otsuka, UCB, Roche and Viatris. C.S. has received speaker’s honoraria from AstraZeneca, Novartis, Fresenius Kabi and BMS; since April 2024, C.S. is an employee of BMS. Other authors declare no conflicts of interest related to this work.
