# Supplementary material for: Ability of SLE-DAS and SLEDAI-2K to predict damage accrual in SLE: a real-world 7-year cohort study from Sweden
Source: Rheumatology (Oxford). 2026 Sep 29;65(10):keag545. doi: 10.1093/rheumatology/keag545 (PMC13661249; doi:10.1093/rheumatology/keag545)
Supplement: Supplementary file 1 [file keag545_supplementary_data.zip › rhe-26-1212-File008.docx]

**Supplementary Table S2:** The predictive value of amSLEDAI-2K, amcSLEDAI-2K, and amSLE-DAS were compared using Akaike Information Criterion (AIC) and Bayesian Information Criterion (BIC). Models for the full cohort (all visits) as well as for 3, 5 and 7 years of follow-up are shown and the lowest AIC and BIC values for each comparison are bolded. Models are adjusted for baseline SDI, age, sex, and mean glucocorticoid dose.

| **All** | | | **Lupus Nephritis** | | | **Arthritis** | | | **Haematological disorder** | | |
| --- | --- | --- | --- | --- | --- | --- | --- | --- | --- | --- | --- |
| ***Full cohort*** | ***AIC*** | ***BIC*** |  | ***AIC*** | ***BIC*** |  | ***AIC*** | ***BIC*** |  | ***AIC*** | ***BIC*** |
| amSLEDAI-2K | 1741.0 | 1756.9 |  | 333.1 | 341.9 |  | 1250.4 | 1264.9 |  | 1015.5 | 1029.1 |
| amcSLEDAI-2K | 1741.7 | 1757.6 |  | 333.2 | 342.1 |  | 1251.5 | 1266.0 |  | 1016.5 | 1030.1 |
| amPhGA | **1738.8** | **1754.6** |  | **330.7** | **339.4** |  | **1247.1** | **1261.6** |  | 1015.2 | 1028.8 |
| amSLE-DAS | 1740.3 | 1756.2 |  | 332.2 | 341.0 |  | 1251.4 | 1265.9 |  | **1014.9** | **1028.5** |
|  |  |  |  |  |  |  |  |  |  |  |  |
| ***3 years*** |  |  |  |  |  |  |  |  |  |  |  |
| amSLEDAI-2K | 787.4 | 798.8 |  | 178.2 | 183.4 |  | 560.4 | 570.4 |  | 490.9 | 500.5 |
| amcSLEDAI-2K | 787.7 | 799.1 |  | 178.2 | 183.4 |  | 560.3 | 570.3 |  | 491.7 | 501.3 |
| amPhGA | 786.5 | 798.0 |  | **173.6** | **178.8** |  | 560.3 | 570.4 |  | 490.3 | 499.9 |
| amSLE-DAS | **783.4** | **794.8** |  | 174.3 | 179.6 |  | **559.8** | **569.9** |  | **488.5** | **498.0** |
|  |  |  |  |  |  |  |  |  |  |  |  |
| ***5 years*** |  |  |  |  |  |  |  |  |  |  |  |
| amSLEDAI-2K | 928.0 | 940.4 |  | 209.1 | 215.4 |  | 666.4 | 677.5 |  | **567.7** | **578.2** |
| amcSLEDAI-2K | 931.4 | 943.8 |  | 209.6 | 215.4 |  | 669.9 | 681.0 |  | 569.6 | 580.1 |
| amPhGA | **926.9** | **939.4** |  | **206.0** | **212.3** |  | **663.5** | **674.6** |  | 568.9 | 579.4 |
| amSLE-DAS | 927.5 | 940.0 |  | 208.8 | 215.0 |  | 670.5 | 681.5 |  | 569.5 | 580.0 |
|  |  |  |  |  |  |  |  |  |  |  |  |
| ***7 years*** |  |  |  |  |  |  |  |  |  |  |  |
| amSLEDAI-2K | 897.7 | 910.2 |  | 229.6 | 236.6 |  | 618.4 | 629.3 |  | 538.3 | 548.9 |
| amcSLEDAI-2K | 900.7 | 912.7 |  | 229.5 | 236.5 |  | 621.5 | 632.4 |  | 540.0 | 550.6 |
| amPhGA | **896.4** | **908.9** |  | **227.3** | **234.3** |  | **616.1** | **627.0** |  | 537.3 | 547.9 |
| amSLE-DAS | 896.6 | 909.1 |  | 229.5 | 236.6 |  | 619.7 | 630.6 |  | **537.0** | **547.5** |

amSLEDAI-2K, adjusted mean SLE Disease Activity Index 2000; amcSLEDAI-2K, adjusted mean clinical SLEDAI-2K; amSLE-DAS, adjusted mean SLE Disease Activity Score; amPhGA, adjusted mean Physician’s Global Assessment.
