# Supplementary material for: Ability of SLE-DAS and SLEDAI-2K to predict damage accrual in SLE: a real-world 7-year cohort study from Sweden
Source: Rheumatology (Oxford). 2026 Sep 29;65(10):keag545. doi: 10.1093/rheumatology/keag545 (PMC13661249; doi:10.1093/rheumatology/keag545)
Supplement: Supplementary file 1 [file keag545_supplementary_data.zip › rhe-26-1212-File007.docx]

**Supplementary Table S1.** Proportions of patients attaining *Definition Of Remission In SLE* (DORIS) remission at follow-up timepoints and across clinical subgroups.

| **Follow-up**  **time** | **Full cohort**  % (*n*/N) | **Lupus Nephritis**  % (*n*/N) | **Arthritis**  % (*n*/N) | **Haematological disorder**  % (*n*/N) |
| --- | --- | --- | --- | --- |
| Baseline | 43.9 (147/335) | 27.1 (23/85) | 43.5 (113/260) | 41.4 (87/210) |
| 1 year | 56.1 (180/321) | 42.7 (35/82) | 55.4 (139/251) | 54.7 (110/201) |
| 3 years | 67.6 (194/287) | 57.1 (44/77) | 66.4 (150/226) | 65.2 (116/178) |
| 5 years | 71.7 (177/247) | 63.8 (44/69) | 71.6 (139/194) | 70.1 (108/154) |
| 7 years | 73.5 (150/204) | 66.7 (38/57) | 72.0 (113/157) | 74.0 (91/123) |
